# Precision treatment of ventilator-induced lung injury through alveolar epithelial cell targeted lipid nanoparticle delivery

**DOI:** 10.7150/thno.111200

**Published:** 2025-05-25

**Authors:** Ning Ding, Hui Xiao, Huiqing Li, Zengzhen Zhang, Junke Ge

**Affiliations:** 1 Hainan Women and Children's Medical Center, Hainan Medical University, Hainan Academy of Medical Sciences, Haikou, China.; 2 Shandong Provincial Key Medical and Health Laboratory of Intensive Care Rehabilitation, Jinan, China.; 3 Department of Anesthesiology, Shandong Provincial Third Hospital, Cheeloo College of Medicine, Shandong University, Jinan, China.; 4 Department of Intensive Care Medicine, Shandong Provincial Third Hospital, Cheeloo College of Medicine, Shandong University, Jinan, China.

**Keywords:** drug delivery, lipid nanoparticle, peptide, ventilator-induced lung injury, cytokine

## Abstract

**Rationale:** Biotrauma characterized by the release of inflammatory cytokines is a key pathological basis of ventilator-induced lung injury (VILI). Small interfering RNA (siRNA) can effectively reduce the release of inflammatory cytokines by inhibiting corresponding inflammatory pathways but may also affect innate immune responses. Therefore, it is promising to target ventilation-induced cytokine production without impairing lung innate immunity.

**Methods:** We developed a novel approach to identify peptide targeting activated alveolar epithelial cells (AECs) in VILI mice by incorporating *in vivo* phage display, high-throughput sequencing, and bioinformatics analysis, and identified a pentapeptide (SPFPT) with high affinity for activated AECs. The SPFPT peptide was then conjugated into lipid nanoparticles (LNPs) to co-deliver importin-7 siRNA (siImp7) and polydatin (PD). The delivery efficiency and biological activity of SPFPT@siImp7/PD-LNP were assessed by *in vitro* and *in vivo* experiments.

**Results:** SPFPT@siImp7/PD-LNP demonstrated significant enhancement in targeting mechanical stretch-activated AECs both *in vitro* and *in vivo*. Intratracheal administration of SPFPT@siImp7/PD-LNP effectively inhibited the release of inflammatory cytokines and ameliorated VILI and associated distal organ injury by simultaneously suppressing p38 and NF-κB pathways. Importantly, SPFPT@siImp7/PD-LNP did not interfere with lung innate immunity.

**Conclusions:** The results suggest that the nanocomplex of SPFPT@siImp7/PD-LNP is promising to be highly effective in the precise treatment of VILI.

## Introduction

Acute lung injury/acute respiratory distress syndrome (ALI/ARDS) is a common cause of respiratory failure, occurring in one-quarter of critically ill patients who require mechanical ventilation 1. Despite advances in supportive care, ALI/ARDS is still associated with substantial mortality and long-term morbidity among survivors 2. The cause of death is more common in multiple organ failure (MOF) than respiratory failure. This deterioration is assumed to be secondary to the involvement of extrapulmonary organs, due to the complex interaction between inflammatory mediators and sustained damage caused by mechanical ventilation (MV) 3. Despite its life-saving properties, MV can exacerbate ALI/ARDS or even worsen outcomes in patients with previously healthy lungs, known as ventilator-induced lung injury (VILI) 4. The biological response of the lungs to MV is referred to as “biotrauma” - the release of inflammatory cytokines that exacerbate lung damage and cause systemic inflammatory responses and MOF 5. Therefore, preventing biotrauma can alleviate MOF and improve survival 6.

The alveolar epithelium forms a continuous lining of the alveolar air spaces and is exposed to large deformations during MV. MV-induced lung overdistension can directly damage alveolar epithelial cells (AECs), or indirectly through the mechanotransduction process that leads to the activation of inflammatory pathways 7-9. We recently reported that importin7 (Imp7) is a p38 interacting protein. Imp7 siRNA (siImp7) inhibited mechanical stretch-induced p38 nuclear translocation and cytokine production 10. Polydatin (PD, 3,4,5-trihydroxystilbene-3-β-D-glucoside), extracted from the roots of Polygonum cuspidatum, possesses anti-inflammatory activity mainly by inhibiting the NF-κB pathway 11. Due to the synergistic effect of the p38 and NF-κB pathways on cytokine production in biotrauma, as well as the potential impairment of innate immunity by blocking p38 and NF-κB, simultaneously inhibiting the activation of p38 and NF-κB induced by mechanical stretch without affecting innate immunity is an effective strategy for treating VILI 12.

The simultaneous delivery of drugs and siRNA using lipid nanoparticles (LNPs) has shown significant advantages in terms of synergistic effects 13. Typically, LNPs are not specific for cells/tissues, so delivering therapeutic agents to certain sites has always been challenging. This can be achieved by incorporating peptide or protein coatings 14. The surface markers of effector cells are closely related to corresponding pathological states or diseases. These molecules are the preferred targets for designing disease-specific delivery of therapeutic agents 15. *In vivo* phage display is an effective technique for identifying peptides that bind to previously unknown targets 16. Given that the phage display library is administered intratracheally in the present study, the selected peptide should bind to molecules located on the apical side of the AECs and be deemed an appealing tool for designing a cell-specific LNP delivery system through inhalation administration.

To develop a pulmonary delivery system for VILI targeted therapy, we presented a novel systematic approach incorporating *in vivo* phage display technique, high-throughput sequencing, and bioinformatics analysis, and identified a pentapeptide (SPFPT) with high affinity for activated AECs in VILI mice. The SPFPT peptide was conjugated to the surface of LNPs to co-deliver siImp7 and PD. The peptide-modified LNPs, SPFPT@siImp7/PD-LNPs, were able to accumulate at activated AECs in VILI mice, and the targeting efficiency, anti-inflammatory action, protective effect against VILI and associated distal organ injury, and impact on lung innate immunity were evaluated both* in vitro* and *in vivo*. Our study provides a novel peptide-modified LNP co-delivery strategy for targeted therapy of VILI.

## Materials and Methods

### Animals

The specific pathogen-free (SPF) C57BL/6 mice were 8-12 weeks old with a body weight of 23-35 g. The animals were housed in laminar flow cages within an SPF facility and were subjected to a 12-hour light-dark cycle while having free access to standard laboratory chow and water. The handling and care of animals were approved by the Animal Ethics Committee of Shandong Provincial Third Hospital, Shandong University (SYDW-2023013).

### VILI model

The VILI mouse model was prepared as previously described 10. In short, the mice received an intraperitoneal injection of an anesthesia mixture containing ketamine (80 mg/kg) and xylazine (10 mg/kg). A tracheostomy was performed, and a sterile catheter was inserted. The mice were connected to a small animal ventilator (Inspira; Harvard Apparatus, Holliston, MA) and ventilated for 4 hours in volume control mode. The ventilation parameters were tidal volume of 20 ml/kg, respiratory rate of 90 breaths/min, and FiO_2_ of 21%. The sham-operated mice underwent the same procedure, but without mechanical ventilation.

### *In vivo* phage display screening

To identify peptides target activated AECs in VILI mice, a Ph.D.-C7C phage library (New England Biolabs, Beverly, MA) was used for *in vivo* screening. Four rounds of selection were performed. In the first round (R1), sham-operated mice were intratracheally injected with 1×10^9^ transducing units (TU) of phages in 50 μL phosphate-buffered solution (PBS) using a MicroSprayer Aerosolizer connected to a high-pressure syringe and a small animal laryngoscope (PennCentury, Wyndmoor, PA). After 30 min, the lungs were lavaged to remove unbound phages, then excised and homogenized. Lung-bound phages were recovered by ER2738 and amplified before the next round of screening. After four rounds of screening, 1000 phage clones were randomly picked, and DNA inserts were amplified and sequenced. In each round, the rescued phages were amplified with ER2738. The titer of amplified phages was measured according to the manufacturer's manual.

### Peptide sequencing

To determine the amino acid sequences displayed by the selected phages, the inserts of the phage were amplified by PCR with the primer: forward, 5'-TGT CGG CGC AAC TAT CGG TAT CAA-3', reverse, 5'-TAG CAT TCC ACA GAC AGC CCT CTA-3'. The PCR products were sequenced on a MiSeq instrument (Illumina, San Diego, CA). The peptide sequence was translated from the DNA sequence.

### Bioinformatic analysis of peptide sequences

The number of occurrences of each tripeptide sequence in a heptapeptide was designated as its abundance. The significance of tripeptide abundance was evaluated using shuffling algorithm and random permutation test (RPT), and the significance level was adjusted using Bonferroni correction. A corrected tripeptide abundance with *p* < 0.05 was considered significant. Multiple sequence alignment was performed using Clustal W.

### Peptide synthesis

Peptides were synthesized via standard Fmoc method using a peptide synthesizer (Peptron Inc., Daejeon, Korea) and characterized by high-performance liquid chromatography (HPLC) and mass spectrometry (Bruker, Bremen, Germany). When included, fluorescein was incorporated into the N-terminus during synthesis. Peptide with a purity of 95% was used in the experiment.

### Affinity analysis of selected phages

Cell-based ELISA was performed to determine the affinity of selected phages to AECs. Mice primary AECs were isolated as previously described 17. AECs were seeded onto a collagen I-coated BioFlex culture plate (Flexercell, McKeesport, PA) and subjected to cyclic stretch on the Flexercell Tension Plus system (FX-4000T; Flexercell, McKeesport, PA) as previously reported 10. Control BioFlex plates with static cells were placed in the same incubator. After gentle washing with Dulbecco's phosphate-buffered saline (DPBS), cells were treated with 0.3% hydrogen peroxide in DPBS for 10 min and blocked with 1% BSA in DPBS. Subsequently, the cells were incubated with candidate or control phages in PBSB for 2 h. After washing, the bound phages were probed using anti-M13/HRP (1:3,000; Sino Biological, Beijing, China) and detected with 3,3',5,5'-tetramethylbenzidine (TMB). The absorbance was read at 450 nm on a microplate reader (Bio-Tec Instruments, Winooski, VT).

### Preparation of siImp7/PD-LNP

SiImp7/PD-LNP was prepared as previously described 18. Briefly, DOTAP (N-[1-(2,3-Dioleyloxy)propyl]-N,N,N-trimethylammonium chloride), cholesterol, polydatin, and DSPE-PEG2000 were dissolved in absolute ethanol in a molar ratio of 1:0.77:0.4:0.03. The organic solvent was evaporated under reduced pressure at 42 °C to form a thin film using a vacuum rotary evaporator (Xi'an HEB Biotechnology, Xi'an, China). RNase-free H_2_O was added to hydrate the lipids and homogenized by ultrasound. The dispersion was extruded through a 0.2 nm Track-Etched membrane (Whatman, Clifton, NJ). Spermidine was added to Imp7 siRNA solution (20 μM) with an N/P ratio of 3:1 and incubated at room temperature for 5 min to form a siRNA/spermidine complex. Then, the mixture was incubated with the prepared LNP dispersion (DOTAP/siRNA = 19/1, weight ratio) at room temperature for 10 min.

### Conjugation of peptide to LNP

The conjugation of peptides to LNP was achieved through carbodiimide reaction between the amine group of the peptide and the carboxyl group of DSPE-PEG2000-COOH, as previously reported 19. In brief, for a 5 mL LNP formulation, 75 mg (w/v) cholesterol, 25 mg (w/v) oleic acid, 50 mg (w/v) PL-90G, 40 μL DSPE-PEG2000-COOH (0.2 mM), 10 μL EDC (200 mM), and 10 μL NHS (200 mM) were added to an ethanol solution and put under vortex movement until clear. The lipid solution was added dropwise to PF-68 solution (12 mL) with stirring. After 30 min, the 0.4 mM peptide solution prepared in PBS at pH 7.4 was added and gently stirred overnight to achieve conjugation. Excess peptides were separated from distilled water using a dialysis membrane (MW 12,000 Da; Sigma, Shanghai, China). The conjugation efficiency (CE) of peptides was quantified by measuring the fluorescence of fluorescein-labeled peptides.

### Characterization of LNPs

The mean particle size, polydispersity index (PDI), and zeta potential of LNP were detected using Zetasizer Nano ZS (Malvern Instruments, Malvern, UK). The surface morphology and shape of LNP were observed under a transmission electron microscope (Hitachi, Tokyo, Japan). The content of polydatin in LNP was measured using a UV-Vis spectrophotometer (Shimadzu, Kyoto, Japan) at 320 nm. The entrapment efficiency (EE) was calculated as follows: EE% = (mass of loaded drug / total mass of added drug) × 100%. The conjugation of peptide to LNP was determined using the CBQCA protein quantitation kit (Molecular Probes, Eugene, OR). The conjugation efficiency (CE%) was calculated as follows: CE% = (amount of peptide conjugated on the LNP surface / total amount of added peptide) × 100%. Quant-iT RiboGreen RNA Assay Kit (Thermo Fisher, Waltham, MA) was used to quantify siRNA encapsulated in the LNP according to the manufacturer's instructions.

### Immunofluorescence

Cells were fixed with 2% (w/v) paraformaldehyde, permeabilized with 0.1% Triton X, and blocked with 2% bovine serum albumin (BSA) in PBS. Then, the samples were incubated with primary antibodies specific for Imp7 (1:200, Thermo Fisher, Rockford, IL), p-p38 (1:200, Cell Signaling, Danvers, MA), and F-actin (1:500, Invitrogen, San Diego, CA). Samples were washed with PBS containing 0.5% BSA followed by incubation in appropriate Cy3 (1:1000, Invitrogen, Carlsbad, CA) and Cy5 (1:1000, Jackson ImmunoResearch Laboratories, West Grove, PA) conjugated secondary antibodies, and stained with 100 ng/ml DAPI (Sigma-Aldrich, St. Louis, MO,). The lung, liver, gut, and spleen were removed and fixed in 4% paraformaldehyde, embedded in paraffin, and sectioned at 4-μm thickness. After incubation with high-affinity probe for F-actin rhodamine phalloidin (Invitrogen, San Diego, CA), the samples were washed with PBS containing 0.5% BSA. Images were obtained using a confocal microscope (FluoView 1000; Olympus, Melville, NY).

To evaluate the binding affinity between LNPs and AECs, Cy5-siImp7 modified LNPs (siImp7/PD-LNP, SPFPT@siImp7/PD-LNP, and a scrambled peptide coupled nanoparticle GTCRV@siImp7/PD-LNP) were incubated with AECs, 3T3, THP-1, and Hela cells in serum-free medium at 37 °C for 4 h. The final concentration of Cy5-siImp7 modified LNPs in the culture medium was 50 nM. For *in vivo* analysis of binding affinity of peptide-modified LNPs, VILI or sham animals were intratracheally administered with Cy5-siImp7 modified LNPs (siImp7/PD-LNP, SPFPT@siImp7/PD-LNP, and GTCRV@siImp7/PD-LNP) at a dose of 5 μg. After 2 h, the lungs were lavaged with PBS and removed for immunofluorescence staining. For quantification, the RGB images were adjusted to the maximum entropy threshold and analyzed using Image J software (NIH Image, Bethesda, MD).

### NF-κB (p65) DNA-binding activity

The nuclear fraction of cell lysates was obtained using a nuclear protein extraction reagent (Thermo Fisher, Rockford, IL), and the NF-κB (p65) DNA-binding activity in a sample containing 5.0 mg of total protein was determined using the NF-κB (p65) DNA-binding TransAM ELISA kit (Thermo Fisher, Rockford, IL). Optical density (OD) was measured at 450 nm.

### Western blotting

Cells were washed with PBS and then exposed to radio immunoprecipitation assay (RIPA) lysis buffer [supplemented with 1 mmol/L phenylmethylsulfonyl fluoride (PMSF)]. A bicinchoninic acid (BCA) protein analysis kit (Beyotime, Shanghai, China) was used to quantify protein concentration. Equal protein amounts were separated by SDS-PAGE followed by immunostaining with primary antibodies against p38 (1:1000), p-p38 (1:2000), p-ATF2 (1:1000), p-MK2 (1:1000), p-IκBα (1:1000), IκBα (1:1000), p-P65 (1:1000), P65 (1:1000). GAPDH was used as internal standard. Horseradish peroxidase conjugated secondary antibodies were used in a standard enhanced chemiluminescence reaction according to manufacturer's instructions (ZSGB, Beijing, China).

### Quantitative real-time PCR

Cell total RNA was extracted using RNeasy Mini Kit (Qiagen, Valencia, CA). For each sample, 1 μg RNA was reverse transcribed using the iScript reverse transcription supermix kit (Bio-Rad, Hercules, CA). PCR amplification mixture was prepared using iTaq SYBR Green Supermix with ROX (Bio-Rad, Hercules, CA), and real-time PCR was performed using MX3000p (Stratagene, La Jolla, CA). The quantification of gene expression of tumor necrosis factor-α (TNF-α), interleukin-6 (IL-6), and high mobility group box-1 protein (HMGB1) was normalized to endogenous GAPDH.

### LPS and LTA intratracheal instillation

Mice were anesthetized and intratracheally instilled with saline, SPFPT@siImp7/PD-LNP, siImp7/PD-LNP (50 nM), or budesonide (1 mg/kg). One hour later, mice were administered 5 μg of LPS or 50 μg of LTA (Sigma, St Louis, MO) in saline solution via intratracheal injection. Five hours later, the lungs were lavaged with PBS, and innate immune indexes were examined by ELISA.

### ELISA

The levels of TNF-α, IL-6, and HMGB1 in BALF and cell culture supernatant were measured by ELISA according to the manufacturer's instructions (R&D Systems, Minneapolis, MN). The concentrations of IgM, lipopolysaccharide-binding protein (LBP), soluble CD14 (sCD14), IL-12, and CXCL5 in BALF were measured using ELISA kits (Abcam, Cambridge, MA). The activity of myeloperoxidase (MPO) in lung samples and diamine oxidase (DAO) in serum were determined using commercially available assay kits (Jiancheng, Nanjing, China).

### Evaluation of tissue inflammation and histological injury

The lungs were weighed immediately after removal, followed by a 48-hour stint in an oven at 75 °C and weighed again. The wet/dry weight ratio was calculated. Differential cell count in BALF was performed using Wright-Giemsa staining. The number of polymorphonuclear neutrophils (PMNs) was determined by a laboratory technologist blind to the experiment to obtain the percentage of neutrophils. Total protein concentration in BALF was examined using a BCA protein assay (Beyotime, Shanghai, China). Serum alanine aminotransferase (ALT) level was measured using a chemistry analyzer (Dri-Chem 7000; Fujifilm, Tokyo, Japan). For histological examination, specimens of fresh lung, liver, and gut were fixed in 10% formalin, embedded in paraffin, serially sectioned, and stained with hematoxylin and eosin (H&E) for light microscopy. A pathologist expert blinded to the experiments evaluated the severity of tissue damage using histological scores of lungs, liver, or gut based on previously described methods 20-22. To investigate the long-term safety of SPFPT@siImp7/PD-LNP, major organs (lung, liver, gut, kidney, and spleen) were excised from mice on the 21st day after intratracheal administration (50 nM) and sectioned for H&E staining.

### Statistical analysis

All statistical tests were performed using Sigmaplot 14.0 (Systat Software, Point Richmond, CA). Data are expressed as mean ± standard error of the mean (SEM). All data were assessed for parametric assumptions, including normality (Shapiro-Wilk test) and homogeneity of variances (Levene's test), and were evaluated using the one-way ANOVA followed by Tukey's post hoc test for multiple comparisons or the two-tailed unpaired Student's t-test for comparison between two groups. Welch's correction was used for parametric data with unequal variances, followed by Dunnett's T3 post hoc test for multiple comparisons. Statistical significance was established at *p* < 0.05.

## Results

### *In vivo* screening of VILI lung tissue targeting peptides

**Figure 1:**
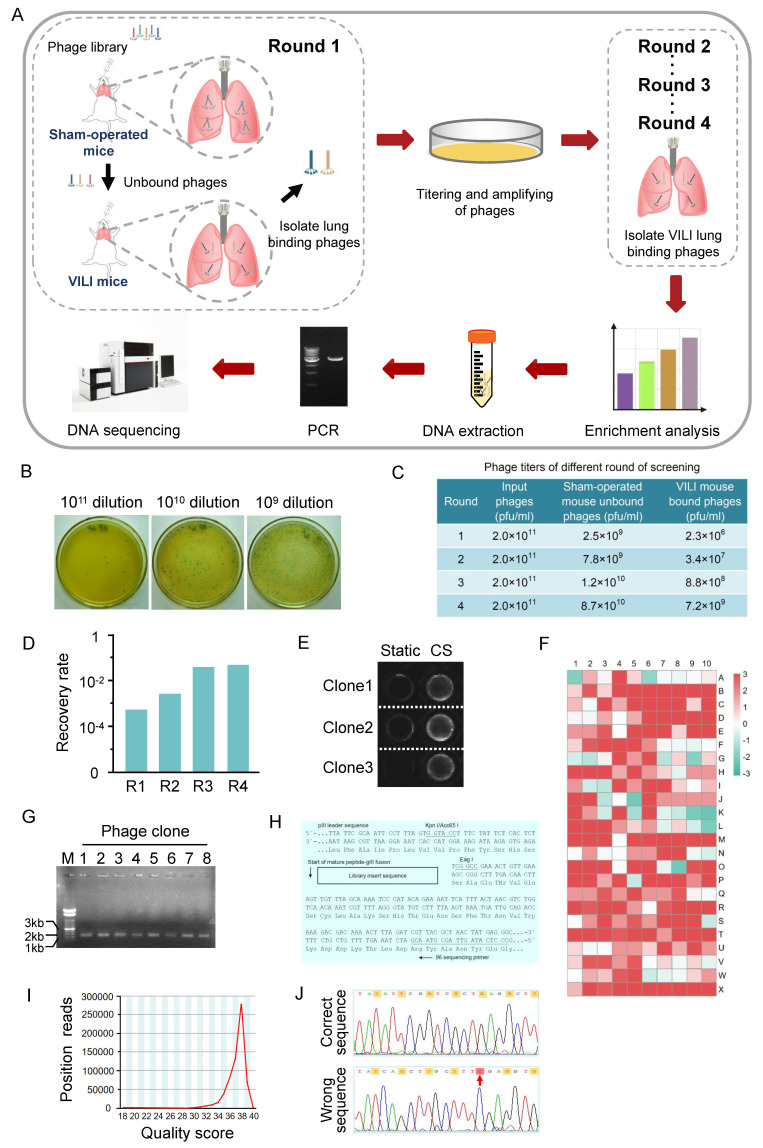
** Screening of peptides target lung tissue of VILI mice.** (A) Flowchart for screening of peptides target lung tissue of VILI mice from a phage-displayed C7C peptide library. The approach was performed through counter-screening against normal lungs (negatively expressing the target molecules) and positive screening against VILI lungs (expressing target molecules) in each round. The phages obtained after four rounds of screening were subject to DNA extraction and PCR amplification. The PCR products containing the sequences encoding heptapeptides were resolved by electrophoresis on 2% agarose and collected for sequencing. (B) An example of phage titer on LB/IPTG/X-gal plates of 10-fold serial dilutions. (C) Titering of phages in different rounds of screening. (D) The recovery rate of phages in different rounds of screening. The recovery rate gradually increased and reached the “saturation point” after four rounds of screening. (E) The affinity of selected phage clones for cyclic stretch (CS) activated-AECs and static AECs was measured by cell-based ELISA. Representative images are shown. (F) Affinity analysis result of different phage clones. According to the standard, the affinity of positive clone for cyclic stretched AECs is three times higher than static AECs. (G) Agarose gel electrophoresis of phage DNA extracted from the positive phages. M: DNA marker; Lane 1~8: DNA of 8 phage clones. (H) The N-terminal sequence of random disulfide-constrained heptapeptide-pIII fusion protein. The fusion protein is expressed with a leader sequence that is removed upon secretion at the position indicated by the arrow, resulting in the alanine preceding the first cysteine residue of the crosslink positioned directly at the N-terminus of the mature fusion protein. The hybridization positions of the -96 primer are indicated. (I) The sequence quality score of PCR products of DNA encoding heptapeptides. The quality score is mainly distributed between 36 and 40. (J) Nucleotide sequences of phage DNA. The design regulation for phage display library oligonucleotides is that the third nucleotide of each codon in the random region should be G or T. The red arrow indicates that codon C does not meet the criterion.

To obtain peptides targeting VILI lung tissue, we used a counter-screening strategy to screen the phage-displayed C7C peptide library. This strategy included an *in vivo* screening of sham-operated mice to eliminate healthy lung adhesives and another screening of VILI lungs. The VILI lung-bound phages were recovered and amplified for the next round of screening. The phages obtained after the fourth round of screening were subject to high-throughput sequencing (Figure 1A).

The effectiveness of the screening strategy is demonstrated by the enrichment of phages after four rounds of screening, which is reflected in the ratio of recovery rates (recovered phages/input phages) between the fourth and the first rounds. In the first round of screening, a starting library (2.0×10^11^ pfu/ml) was given to the sham-operated mice, and the titer of unbound phages collected from the lavage fluid was 2.5×10^9^ pfu/ml. The amount of phage in the lungs and BALF was determined by titering using LB/IPTG/X-gal plate. Phages lyse bacterial lawns on plate to produce plaques, which are used to determine the concentration (titer) of the phages. Phage titer is expressed as the number of plaque forming units (pfu) in a given volume. An example of phage titering on LB/IPTG/X-gal plates of 10-fold serial dilutions is shown in Figure 1B. The phages were administered to the VILI mouse, and the titer of phages bound to the lungs was 2.3×10^6^ pfu/ml. Therefore, the recovery rate of the first round of screening was 9.2×10^-4^. After four rounds of screening, the titer of unbound phages increased in sham-operated mice. The titer of VILI lung-bound phages also increased (Figure 1C). The recovery rate of phages gradually increased and stabilized after four rounds of screening, indicating that phages specifically bound to VILI lungs were effectively enriched and reached the “saturation point” (Figure 1D). Due to the coverage of AECs on the alveolar surface, the binding affinity of each selected phage to AECs was validated. After the final round of screening, 1000 phage clones were randomly selected and their affinity for cyclic stretch (CS) activated-AECs and static AECs was measured by cell-based ELISA. Figure 1E shows a representative image of cell-based ELISA assay. According to the standard that positive clones have a 3-fold higher affinity for CS activated-AECs than static AECs, 723 out of 1000 phage clones were designated positive phages. Figure 1F shows the result of affinity analysis. Agarose gel electrophoresis indicated that the single-strand DNA extracted from the positive phages was consistent with the size of phage DNA (Figure 1G).

The sequence data generated with the -96gIII sequencing primer corresponded to the anticodon strand of the gIII region of the template. The reverse complement of the data was obtained and compared the result against the top strand of the insert sequence shown in Figure 1H. The sequence quality score was mainly distributed between 36 and 40, indicating good quality of sequencing data (Figure 1I). Due to the design of phage display library oligonucleotides, the third nucleotide of each codon in the random region should be G or T. Sixteen phage clones were excluded because some codons in the random region did not meet the regulation. For example, the third nucleotide of the fifth codon is C in the random region sequence TAT CAG CTT GCT TTC GAG GTG (Figure 1J). Possible reasons could be wild-type phages or sequencing errors. Therefore, a total of 707 heptapeptide sequences were obtained.

### High-throughput analysis of the selected peptide sequences

**Figure 2:**
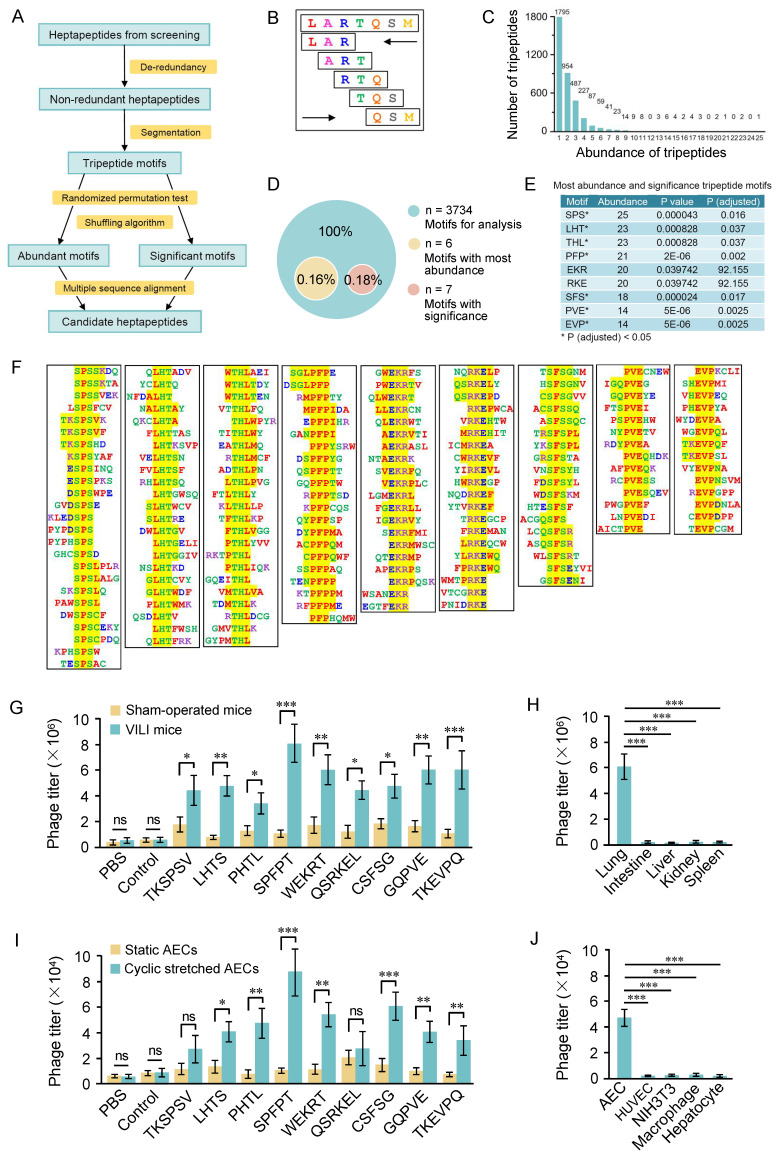
** High-throughput analysis of the selected peptide sequences. (**A) Flowchart of bioinformatic analysis of the selected heptapeptide sequences. (B) Diagram of the segmentation of heptapeptide. The heptapeptide was continuously segmented into five tripeptide motifs in both forward and reverse directions. (C) Abundance and distribution of tripeptide motifs. (D) Composition of the most abundant and significant tripeptide motifs. The abundance of 6 tripeptide motifs was above twenty. Random permutation test (RPT) and Bonferroni correction method were used for significance evaluation and the correction of significance level. Tripeptide with a correction of P < 0.05 was considered significant. (E) The most abundant and significant tripeptide motifs. (F) Identification of extended motifs containing the most abundant and significant tripeptide motifs (SPS, LHT, HTL, PFP, EKR, RKE, SFS, PVE, EVP) with Clustal W analysis. (G) *In vivo* phage binding assay of phages displaying the indicated motifs. The SPFPT phage exhibited the highest binding affinity with VILI lungs, 9.5 times higher than that of the normal lungs and 7.3 times higher than that of the control phage. (H) Tissue distribution of the SPFPT phage was evaluated by *in vivo* phage binding assay. (I) *In vitro* phage binding assay of phages displaying the indicated motifs. The SPFPT phage showed the highest binding affinity with cyclic stretched AECs, 7.1 times higher than that of the static AECs and 8.2 times higher than that of the control phage. (J) Cell binding specificity of the SPFPT phage was evaluated by *in vitro* phage binding assay. Data are expressed as mean ± SEM of triplicate samples. Significance: * p < 0.05, ** p < 0.01, ***p < 0.001, ns: no significance.

We designed a high-throughput strategy to analyze the distribution of inserts in the 707 heptapeptide sequences (Figure 2A). De-redundancy analysis was performed on the 707 heptapeptides using CD-HIT software and 638 non-redundant heptapeptides were obtained. The non-redundant heptapeptides were then continuously segmented into tripeptide motifs in both forward and reverse directions (Figure 2B), and a total of 6380 (5×2×638) tripeptides were obtained. The frequency of occurrence of a specific tripeptide was counted as the abundance of the tripeptide. Figure 2C shows that 6380 tripeptides contained 3734 tripeptides, six peptides with an abundance exceeding 20. Significance evaluation analysis shows that the abundance of 7 tripeptides is significant (Figure 2D). The nine tripeptides with significance or abundance exceeding 20 were selected for further analysis (Figure 2E). ClustalW was then used to analyze the original seven peptide inserts containing these tripeptides, and 4-6 amino acid motifs were obtained (Figure 2F).

Next, we conducted an *in vivo* phage binding assay to determine whether phages displaying the representative motifs specifically bound to VILI lungs. Titers of phages in the VILI lungs were significantly higher than those in the control lungs. The SPFPT phage exhibited the highest binding affinity with VILI lungs, 9.5 times higher than that of the normal lungs and 7.3 times higher than that of the control phage (Figure 2G). Tissue distribution of the SPFPT phage was also evaluated by the titers of phage in multiple organs (lung, gut, liver, kidney, and spleen). The result showed that only the lungs were the target organ (Figure 2H). Further study was conducted on the ability of the phages to target AECs *in vitro*. The SPFPT phage showed the highest binding ability to CS induced AECs (Figure 2I). Cell-specific binding assay confirmed that the SPFPT phage only bound to AECs (Figure 2J). SPFPT exhibited the highest binding activity among the candidate motifs and was therefore selected for further study.

### *In vivo* targeting and cellular localization of SPFPT peptide

**Figure 3:**
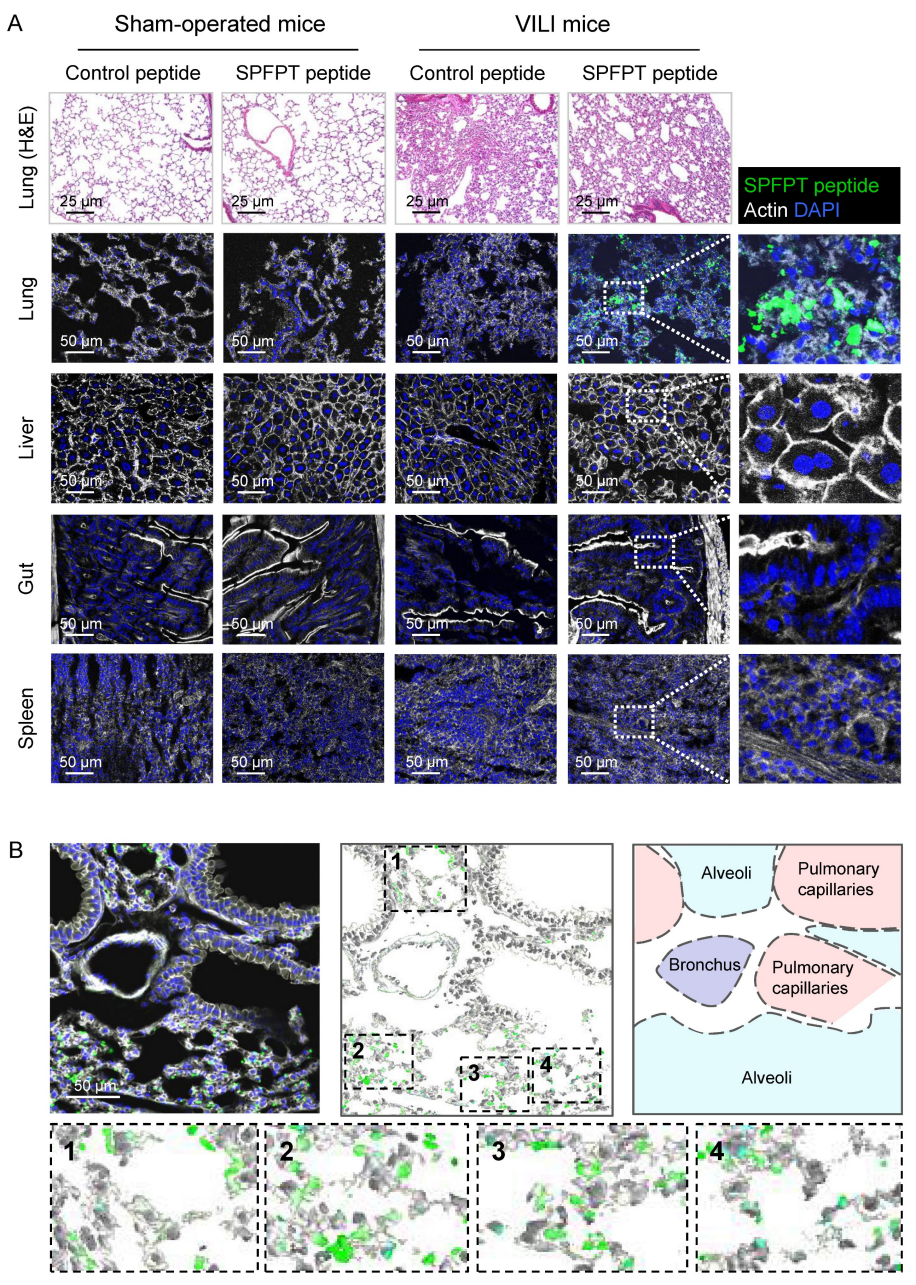
***In vivo* targeting and cellular localization of the SPFPT peptide.** (A) SPFPT peptide targets VILI lung tissue. Fluorescein-conjugated SPFPT peptide and control scrambled peptide were synthesized and intratracheally administered into the lungs of VILI and sham-operated mice. H&E staining showed inflammatory lung injury in VILI mice, while the lungs of sham-operated mice were normal. Immunofluorescence staining showed that SPFPT peptide bound to the lungs of VILI mice, but not sham-operated mice. The SPFPT peptide was not observed in the liver, gut, and spleen. (B) The binding of the SPFPT peptide to AECs in the lungs of VILI mice. Immunofluorescence staining showed that the SPFPT peptide was localized within the alveoli and bound to AECs.

To investigate whether SPFPT mediated the targeting of SPFPT phage to VILI lungs, fluorescein-conjugated SPFPT peptide and control scrambled peptide were intratracheally administered to the mice. Histological staining of lung tissues showed that MV induced marked inflammatory changes (Figure 3A). Immunostaining showed that the SPFPT peptide bound to the lungs of VILI mice but not to sham-operated mice. No binding of the control peptide was observed in the lungs of VILI and sham-operated mice. In organ-specific experiments, the SPFPT peptide was not observed in the liver, gut, and spleen (Figure 3A). The cellular localization of the SPFPT peptide was further examined. Immunostaining showed that the SPFPT peptide was localized within the alveoli, indicating that the SPFPT peptide bound to AECs (Figure 3B).

### Preparation and characterization of SPFPT@siImp7/PD-LNP

**Figure 4:**
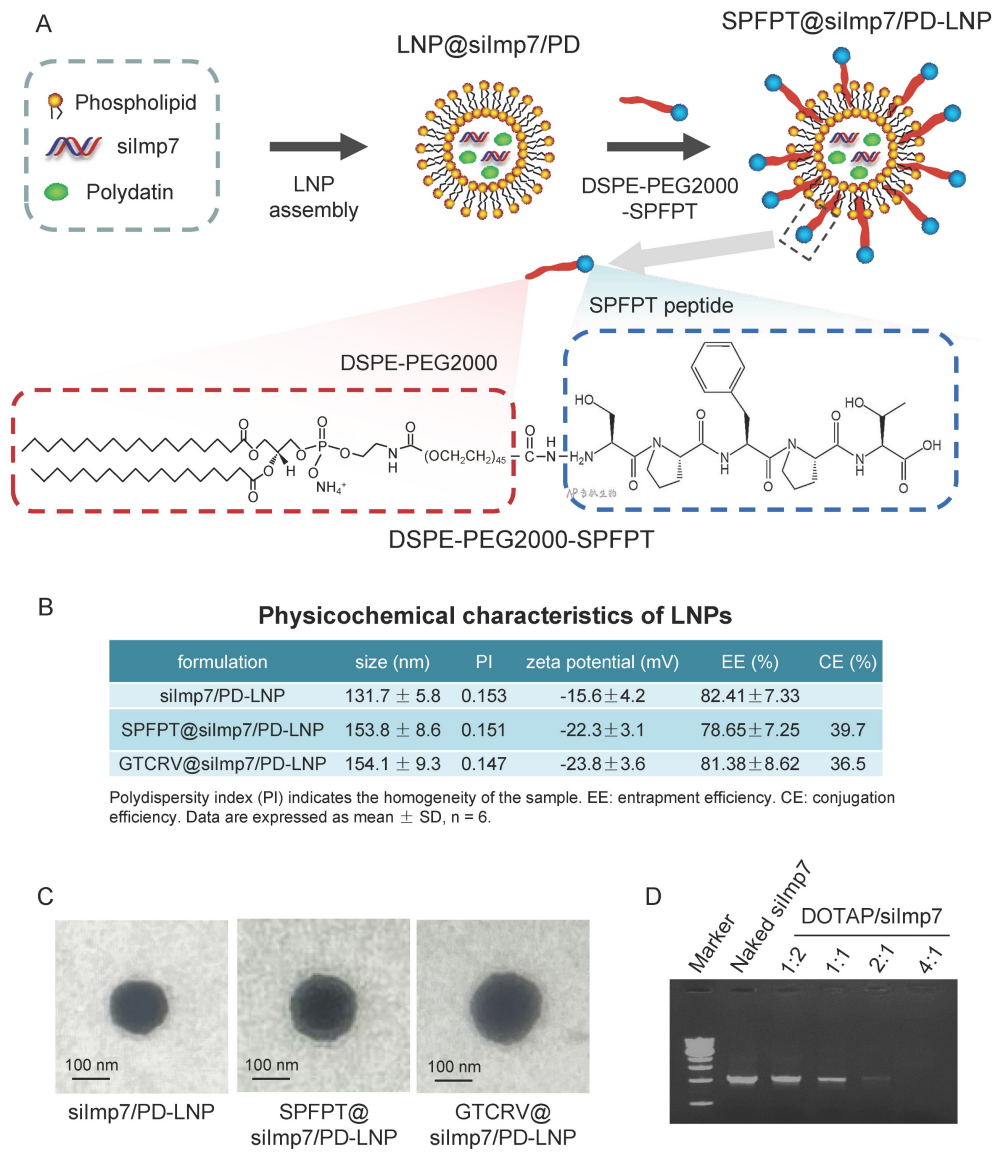
** Preparation and Characterization of SPFPT@siImp7/PD-LNP. (**A) A peptide-modification strategy was utilized to conjugate SPFPT peptide onto LNP surfaces for active targeting. LNP were formulated with phospholipids, DSPE-PEG2000, siImp7, and polydatin. The resulting LNP was further conjugated with SPFPT for targeted LNP formulation. The conjugation of the SPFPT peptide with LNP was achieved through a carbodiimide reaction between the amine group of the peptide and the carboxyl group of DSPE-PEG2000-COOH. (B) The physicochemical characteristics of the LNPs. (C) Scanning electron microscope (SEM) photographs of siImp7/PD-LNP, SPFPT@siImp7/PD-LNP, and GTCRV@siImp7/PD-LNP. The LNPs were regular and spherical in shape, with a smooth exterior and 100-150 nm in size. (D) Agarose gel retardation assay of the LNPs. Electrophoretic bands of siImp7 at various DOTAP/siRNA molar ratios from 1:2 to 4:1.

The schematic structure of the siImp7/PD-LNP shows that the core of the siImp7/PD complex is enveloped in the phospholipid bilayer. The DSPE-PEG2000-SPFPT compound is conjugated to the surface of siImp7/PD-LNP to form targeted nanoparticle SPFPT@siImp7/PD-LNP (Figure 4A). Three LNPs were prepared to encapsulate siImp7 and PD, siImp7/PD-LNP, SPFPT@siImp7/PD-LNP, and scrambled peptide coupled nanoparticle GTCRV@siImp7/PD-LNP. Physicochemical characteristics of the LNPs are summarized in Figure 4B. The average diameter of LNPs was in the range of 130-160 nm, and the polydispersity index (PI) of LNPs was less than 0.2, indicating that the sizes of LNPs were homogeneous. The zeta-potentials of SPFPT@siImp7/PD-LNP and GTCRV@siImp7/PD-LNP were lower than that of siImp7/PD-LNP, indicating the shielding effect of hydrophilic PEG group and peptide moiety on the surface of LNPs. The success of the peptide coupling process was shown by the subsequent increase in particle size and drop in zeta potential following peptide conjugation.

There was no significant difference of entrapment efficiency between siImp7/PD-LNP and SPFPT@siImp7/PD-LNP or GTCRV@siImp7/PD-LNP, indicating the addition of peptide did not alter the parameter. The morphology of the LNPs was examined by scanning electron microscope (SEM) (Figure 4C). It appeared that the LNPs were regular and spherical in shape, with a smooth exterior and 100-150 nm in size. The slightly larger particle sizes of the SPFPT@siImp7/PD-LNP and GTCRV@siImp7/PD-LNP were likely due to the SPFPT or GTCRV peptide coating. Gel retardation assay showed that the decrease in the band signal of siImp7 was accompanied by an increase in the DOTAP/siRNA ratio. The signal nearly disappeared when the ratio was 2:1, indicating that siImp7 was mostly encapsulated in LNPs (Figure 4D).

### Enhanced AECs delivery efficiency via SPFPT-conjugated LNPs

**Figure 5:**
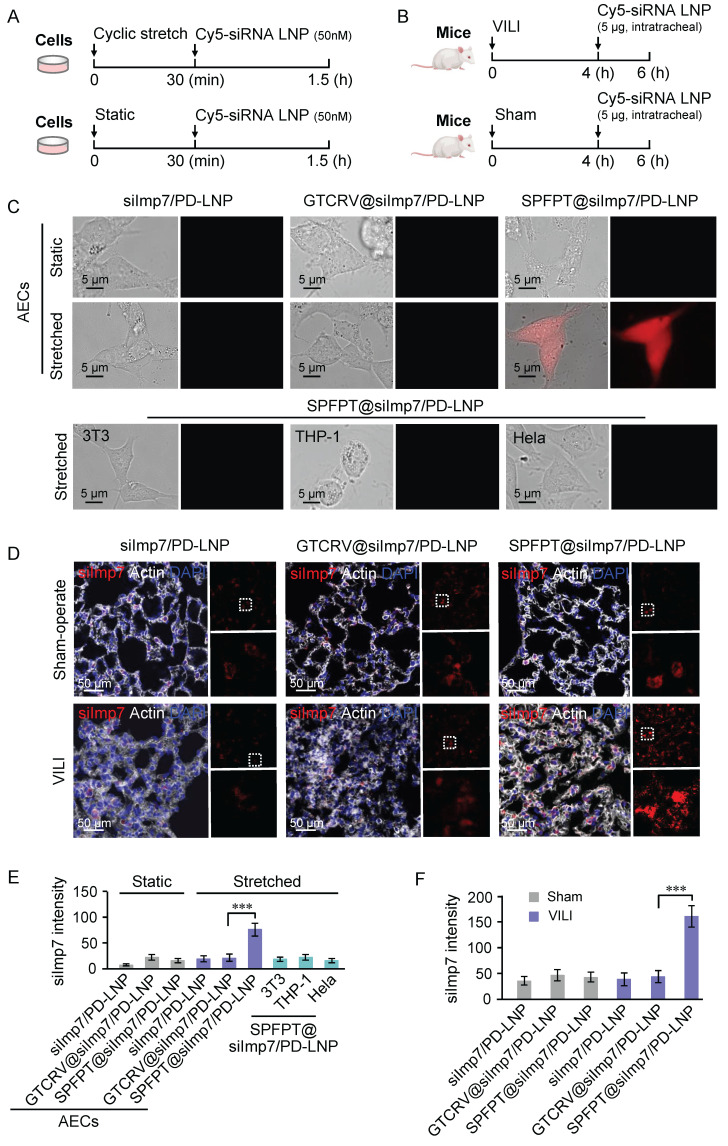
** SPFPT coupling enhances the delivery efficiency of LNPs in AECs. (**A) Timeline for transfection with Cy5-labeled siImp7/LNPs *in vitro*. AECs were subjected to cyclic stretch. Static cells served as control. After 30 min, the cells were incubated with Cy5-labeled siImp7/LNPs (siImp7/PD-LNP, SPFPT@siImp7/PD-LNP, and GTCRV@siImp7/PD-LNP) for 1 h, and fluorescence microscopy was used to evaluate siImp7 expression. (B) Timeline for Cy5-labeled siImp7/LNPs treatment *in vivo*. C57BL/6 mice were mechanically ventilated for 4 h. The sham-operated mice served as controls. The animals were then intratracheally administered with Cy5-labeled siImp7/LNPs (siImp7/PD-LNP, SPFPT@siImp7/PD-LNP, and GTCRV@siImp7/PD-LNP). After 2 h, the lungs were lavaged and samples were examined by fluorescence microscopy. (C) Representative images of siImp7 expression in static and cyclic stretched AECs, and cyclic stretched 3T3, THP-1, and Hela cells under the microscope (Scale bar, 5 μm). (D) Representative images of siImp7 expression in the lungs of VILI and sham-operated mice under the microscope. (Scale bar, 50 μm). (E) Quantification of siImp7 intensity in static and cyclic stretched AECs, and cyclic stretched 3T3, THP-1, and Hela cells. (F) Quantification of siImp7 intensity in the lungs of VILI and sham-operated mice. Data are expressed as mean ± SEM of triplicate samples. Significance: ***p < 0.001.

Peptide coupling strategies have been proven to selectively target cell types in various therapeutic applications 23. To evaluate the target specificity and delivery efficiency of SPFPT-coupled LNPs, Cy5-labeled LNPs (siImp7/PD-LNP, SPFPT@siImp7/PD-LNP, and scrambled peptide coupled LNP GTCRV@siImp7/PD-LNP) were used for *in vitro* (Figure 5A) and *in vivo* (Figure 5B) analysis. Compared with GTCRV@siImp7/PD-LNP, SPFPT@siImp7/PD-LNP showed advantages in delivery efficiency in both CS induced-AECs (Figure 5C, E) and VILI mice (Figure 5D, F). No significant difference was observed between GTCRV@siImp7/PD-LNP and siImp7/PD-LNP groups. *In vitro* analysis indicated that SPFPT@siImp7/PD-LNP targeted AECs (Figure 5C, E). *In vivo* experiments confirmed that SPFPT modification significantly enhanced lung targeting and delivery efficiency (Figure 5D, F).

### SPFPT@siImp7/PD-LNP inhibits the production of inflammatory cytokines

**Figure 6:**
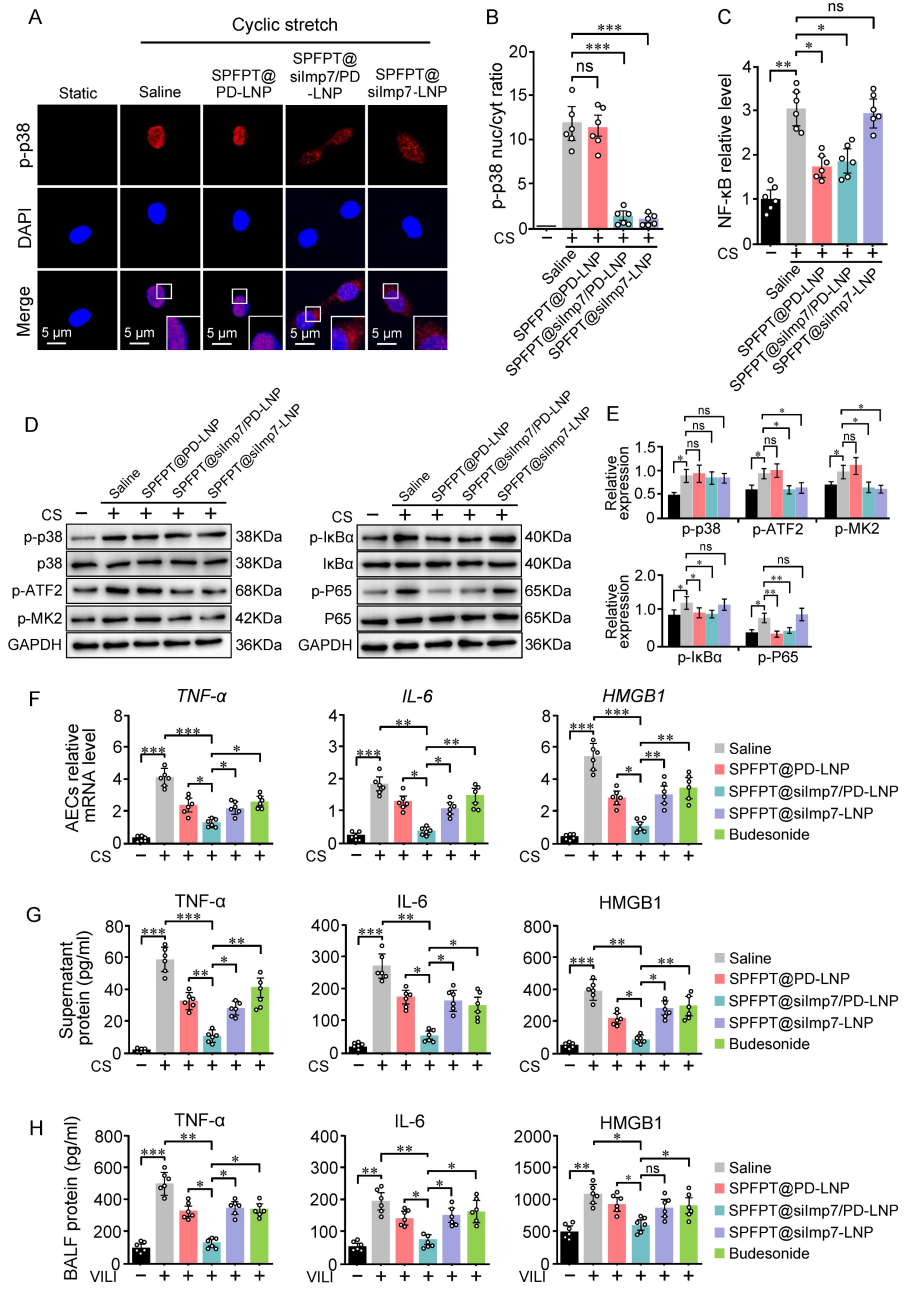
** SPFPT@siImp7/PD-LNP inhibits the release of proinflammatory cytokines.** AECs were incubated with saline, SPFPT@PD-LNP, SPFPT@siImp7/PD-LNP, and SPFPT@siImp7-LNP for 1 h, and then subjected to cyclic stretch. Static cells served as control. **(**A) Representative images of p-p38 expression in AECs under the microscope (Scale bar, 5 μm). Immunostaining was performed with p-p38 (red) and counterstained with DAPI to detect nuclei (blue). (B) Quantification of the nucleo-cytoplasmic distribution of p-p38. (C) Relative NF-κB activity in CS-stimulated AECs incubated with saline, SPFPT@PD-LNP, SPFPT@siImp7/PD-LNP, and SPFPT@siImp7-LNP. (D) Western blotting analysis of p-p38, p38, p-ATF2, p-MK2, p-IκBα, IκBα, p-P65, P65, and GAPDH in AECs. (E) Relative expression of p-p38, p-ATF2, p-MK2, p-IκBα, and p-P65 in AECs measured by Western blotting. (F) AECs were incubated with saline, SPFPT@PD-LNP, SPFPT@siImp7/PD-LNP, SPFPT@siImp7-LNP, and budesonide for 1 h, and then subjected to cyclic stretch. Static cells served as control. The mRNA levels of TNF-α, IL-6, and HMGB1 in AECs were evaluated using qRT-PCR. (G) AECs were incubated with saline, SPFPT@PD-LNP, SPFPT@siImp7/PD-LNP, SPFPT@siImp7-LNP, and budesonide for 1 h, and then subjected to cyclic stretch. Static cells served as control. The protein levels of TNF-α, IL-6, and HMGB1 in the culture supernatant of AECs were evaluated using ELISA. (H) Mice were randomized to the sham-operated group and the VILI group. In the VILI group, mice were intratracheally administered with saline, SPFPT@PD-LNP, SPFPT@siImp7/PD-LNP, SPFPT@siImp7-LNP, and budesonide, respectively. The protein levels of TNF-α, IL-6, and HMGB1 in BALF of mice were evaluated using ELISA. Data are expressed as mean ± SEM from 6 mice per group. Significance: * p < 0.05, ** p < 0.01, ***p < 0.001, ns: no significance.

SPFPT@siImp7/PD-LNP inhibited p38 nuclear import and NF-κB activation in CS-stimulated AECs, indicating the feasibility of the targeted delivery approach (Figure 6A-C). Western blotting results confirmed that SPFPT@silmp7/PD-LNP simultaneously suppressed p38 and NF-κB pathways (Figure 6D, E). SPFPT@siImp7/PD-LNP exhibited higher inhibitory effect on the mRNA levels of TNF-α, IL-6, and HMGB1 in CS-stimulated AECs than the SPFPT@PD-LNP and SPFPT@siImp7-LNP (Figure 6F). ELISA analysis indicated that CS induced the release of TNF-α, IL-6, and HMGB1 into the culture supernatant. SPFPT@siImp7/PD-LNP significantly decreased the levels of these cytokines (Figure 6G). The animal experiments confirmed that the SPFPT@siImp7/PD-LNP most significantly reduced the levels of TNF-α, IL-6, and HMGB1 in BALF (Figure 6H). We further compared the efficacy of SPFPT@silmp7/PD-LNP with budesonide, a widely used inhaled corticosteroid that had been shown to alleviate lung inflammation in experimental and clinical models 24,25. The results showed that SPFPT@silmp7/PD-LNP was superior to budesonide in inhibiting mRNA and protein levels of these cytokines (Figure 6F-H).

### SPFPT@siImp7/PD-LNP alleviates VILI and associated distal organ injury

**Figure 7:**
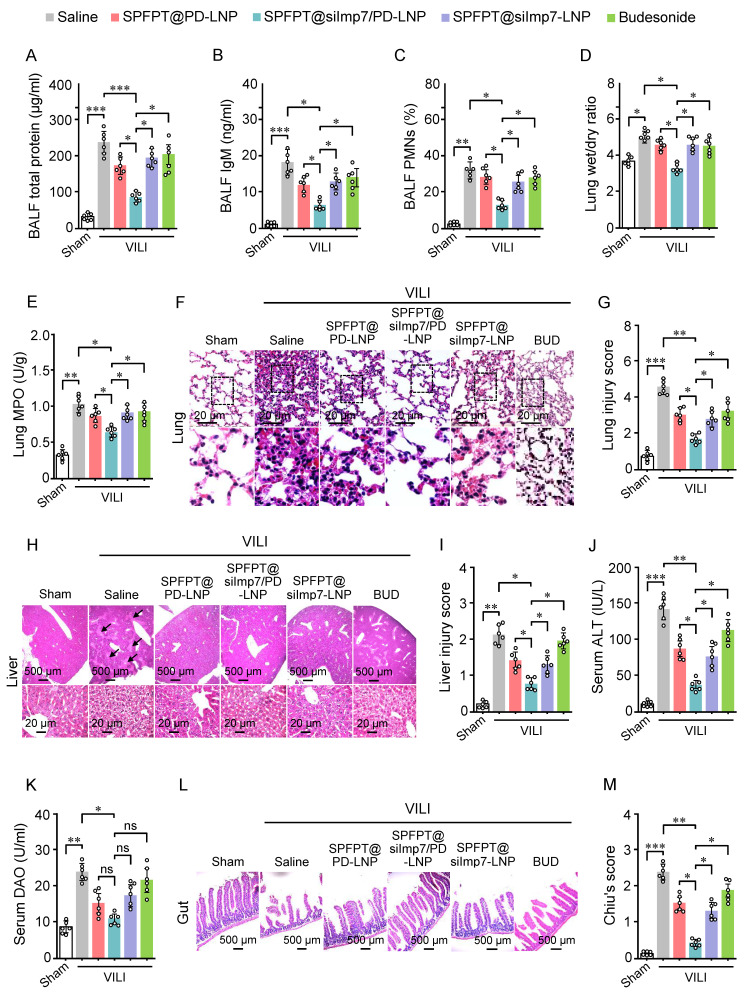
** SPFPT@siImp7/PD-LNP alleviates VILI and associated distal organ injury.** Mice were randomized to the sham-operated group and the VILI group. In the VILI group, mice were intratracheally administered with saline, SPFPT@PD-LNP, SPFPT@siImp7/PD-LNP, SPFPT@siImp7-LNP, and budesonide, respectively. Levels of total protein (A), IgM (B), and the percentage of polymorphonuclear neutrophils (PMN) in bronchoalveolar lavage fluid (BALF) (C), lung wet/dry weight ratio (D), and lung MPO activity (E) were measured. (F) Lung tissue sections were stained with hematoxylin and eosin (H&E). Representative images are shown for each group. (G) Lung damage scores were determined based on leukocyte infiltration, exudative edema, and alveolar wall thickness. (H) Liver tissue sections were stained with H&E. Representative images are shown for each group. Arrows indicate damaged tissue. (I) Injury scoring (Eckhoff's score) of the liver. (J) Serum alanine aminotransferase (ALT) levels. (K) Serum diamine oxidase (DAO) levels. (L) Gut tissue sections were stained with H&E. Representative images are shown for each group. (M) Chiu's score of gut. Data are expressed as mean ± SEM from 6 mice per group. Significance: * p < 0.05, * * p < 0.01, ***p < 0.001, ns: no significance.

The therapeutic effect of SPFPT@siImp7/PD-LNP on VILI mice was investigated. MV induced significant increases in total protein (Figure 7A), IgM (Figure 7B), and the percentage of PMNs (Figure 7C) in BALF that reflects lung inflammation. Lung wet/dry weight ratio (Figure 7D), an indicator of lung edema, and MPO activity (Figure 7E), an index of neutrophil infiltration, were also significantly increased. The lung histopathology (Figure 7F) and damage score (Figure 7G) further confirmed inflammatory injury caused by MV. Treatment with SPFPT@siImp7/PD-LNP showed significant improvement in these inflammatory indexes and lung histology compared to SPFPT@PD-LNP, SPFPT@siImp7-LNP, and budesonide (Figure 7A-G).

Lung-derived cytokines not only exacerbate lung injury but may also spill over into the systemic circulation, affecting extrapulmonary organs such as the liver and gut, ultimately leading to functional impairment of these organs 20,26. We thus evaluated the protective effect of SPFPT@siImp7/PD-LNP on the liver and gut in the VILI mice. VILI mice showed significantly increased liver histological injury (Figure 7H, 7I) and serum ALT level (Figure 7J), indicating the presence of acute liver injury. Compared to the SPFPT@PD-LNP, SPFPT@siImp7-LNP, and budesonide groups, the degree of liver injury and serum ALT levels significantly reduced in the SPFPT@siImp7/PD-LNP group (Figure 7H-J). Serum DAO, a marker of intestinal mucosal disorder, was considerably higher in the VILI mice than in the sham-operated group (Figure 7K). In addition, the intestinal histological injury (Figure 7L) and Chiu's score (Figure 7M) also significantly increased in the VILI mice. Administration of SPFPT@siImp7/PD-LNP showed the best therapeutic effects on these indexes, most probably attributed to the synergistic anti-inflammatory efforts of siImp7 and PD (Figure 7K-M).

### SPFPT@siImp7/PD-LNP does not impair lung innate immunity

**Figure 8:**
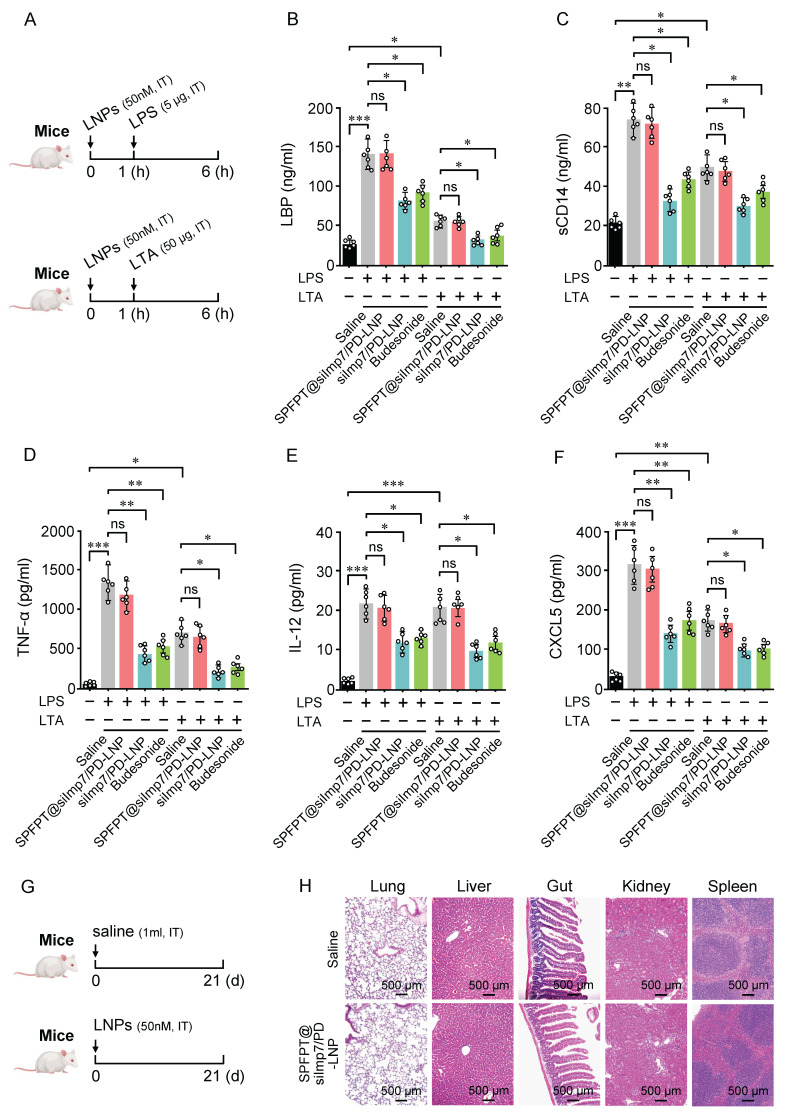
** SPFPT@siImp7/PD-LNP does not interfere with lung innate immunity.** (A) Mice were intratracheally administered with saline, SPFPT@siImp7/PD-LNP, siImp7/PD-LNP, and budesonide respectively. After 1 h, animals were intratracheally administered with LPS (5 μg) or LTA (50 μg). After 5 h, the lungs were lavaged and innate immune indexes were examined. Levels of LBP (B), sCD14 (C), TNF-α (D), IL-12 (E), and CXCL5 (F) in bronchoalveolar lavage fluid (BALF) were measured by ELISA. (G) Mice were intratracheally administered with saline and SPFPT@siImp7/PD-LNP, respectively. On the 21st days, all animals were sacrificed for in vivo safety evaluation. (H) H&E staining images of lung, liver, gut, kidney, and spleen sections. Data are expressed as mean ± SEM from 6 mice per group. Significance: *p < 0.05, **p < 0.01, ***p < 0.001, ns: no significance.

In addition to mediating inflammatory responses, the p38 and NF-κB pathways also play key roles in innate immune response 27,28. Therefore, strategy targeting p38 and NF-κB inhibition may interfere with innate immunity. We next investigated whether SPFPT@siImp7/PD-LNP impacts innate response induced by lipopolysaccharide (LPS) or lipoteichoic acid (LTA) in mouse lungs (Figure 8A). As shown in Figure 8B-C, LPS and LTA elicited significant upregulation of LBP and sCD14 in BALF. SPFPT@siImp7/PD-LNP had a negligible impact on LBP and sCD14 levels, while siImp7/PD-LNP and budesonide significantly decreased the levels of both proteins. TNF-α and Th1 cytokines, such as IL-12, are important mediators of innate immunity, particularly against bacterial pathogens in the lungs 29. In this study, the levels of TNF-α and IL-12 in BALF significantly increased in the LPS or LTA-challenged animals. SPFPT@siImp7/PD-LNP treatment did not significantly affect both proteins (Figure 8D-E). CXCL5, a critical mediator that induces neutrophil infiltration in the pulmonary innate immune response 30, was significantly upregulated in the BALF of LPS or LTA-treated mice. Again, SPFPT@siImp7/PD-LNP did not significantly alter the level of CXCL5 (Figure 8F). To further evaluate the long-term safety of SPFPT@siImp7/PD-LNP, major organs were excised from mice on the 21st day and sectioned for H&E staining (Figure 8G). Compared to the saline group, there were no obvious pathological changes in the lung, liver, gut, kidney, and spleen of the SPFPT@siImp7/PD-LNP group (Figure 8H). These results suggest that SPFPT@siImp7/PD-LNP does not impair the innate immune response of the lung to bacteria and has good biosafety in the animal model.

## Discussion

LNPs provide new opportunities for developing more effective, safe, and commercially viable vehicles for drug delivery. In particular, LNPs offer the possibility of incorporating multiple functionalities within a single construct. The effect can be enhanced by incorporating peptide or protein coatings to improve pharmacokinetics and enable tissue targeting 31. Although specifically expressed receptors are therapeutic targets for certain diseases, identifying targets for peptide binding remains a bottleneck in developing new therapies. To overcome this challenge, we developed a high-throughput strategy that allows for the discovery of binding peptides targeting disease-specific cells/tissues. Significantly, this phage display-based strategy is an unbiased approach that targets peptide-activated cell interaction without any prior knowledge of properties of the interacting partner proteins. By applying this strategy, we successfully identified targeting peptide for activated AECs in VILI mice. The selected SPFPT peptide was conjugated to LNPs to co-deliver siImp7 and PD. *In vitro* and *in vivo* experiments demonstrated that SPFPT@siImp7/PD-LNP accumulated at AECs, and the conjugation of SPFPT improved the delivery efficiency of siImp7/PD. Further analysis revealed that SPFPT@siImp7/PD-LNP inhibited the release of inflammatory cytokines and alleviated VILI and associated distal organ injury. The effects were achieved by inhibiting mechanical stretch-activated p38 and NF-κB in AECs. Importantly, SPFPT@siImp7/PD-LNP did not impair lung innate immunity, underlining the clinical prospects of treating VILI.

Targeted LNPs provide attractive approaches as they may only bind to the desired cells/tissues. This can be achieved by introducing recognition motifs around the corona of the LNP, which can preferentially bind to cell surface receptors and other biomolecules exposed within the target tissue. Short peptides have emerged as the preferred agent for influencing LNP distribution due to several advantageous properties, including lowered immunogenicity, increased stability, and reduced binding to physiological biomolecules compared to full-length proteins 31. A specific challenge in VILI therapy development is the lack of VILI-specific cell surface receptors that are expressed by activated cells of the disease, but not normal cells. To meet this challenge, we took an unbiased approach and selected phage libraries on live VILI lung tissues and cells to identify novel peptides that bind to activated AECs in VILI, but not normal AECs and other normal cells. The approach is entirely unbiased with no prior knowledge of the target receptors 32. This approach was performed through counter-screening against normal lungs (negatively expressing the target molecules) and positive screening against VILI lungs (expressing target molecules). Following four rounds of increasingly stringent screening, peptides with high affinity were identified.

In this study, the tripeptide motif was chosen as the basic unit for phage insert analysis because the three amino-acid residues provide the minimal framework for structural formation and protein-protein interactions 33. Examples of such biochemical recognition units and binding of ligand motifs to their receptors include RGD, LDV, and LLG to integrins 34,35, NGR to aminopeptidase N/CD13 36,37, and GFE to membrane dipeptidase 38,39. After phage display screening, 707 heptapeptide sequences were obtained. To analyze the distribution characteristics of tripeptides in the 707 heptapeptides and obtain specific amino acid motifs containing these tripeptides, we designed a high-throughput strategy that includes de-redundancy analysis, heptapeptide segmentation, RPT, Bonferroni correction, and ClustalW analysis. This analysis revealed 4 to 6 amino acid motifs shared among multiple peptides, with the SPFPT peptide showing the highest affinity. The main reason for successfully identifying peptides is that the selected peptide binds to natural receptors when expressed *in vivo*, even though ligand-receptor interactions are mediated through conformational epitopes rather than linear epitopes 40. Therefore, the peptides selected *in vivo* may be more suitable for clinical applications.

AECs release inflammatory cytokines when exposed to cyclic stretch during mechanical ventilation 41. Due to the critical role of cytokines such as TNF-α, IL-6, and HMGB1 in VILI, regulating cytokines release to control inflammation may treat VILI and associated distal organ damage characterized by cytokine storm 10,20,26. P38 MAPK plays a crucial role in transducing mechanical signals during VILI. In response to mechanical forces, p38 undergoes phosphorylation and then translocated to the nucleus, inducing gene transcription of pro-inflammatory mediators 10. Imp7 is a newly discovered p38 interacting protein that facilitates CS-induced p38 nuclear import and is a novel target for VILI therapy 10. NF-κB is another critical signaling pathway that triggers mechanical stretch-induced inflammatory response 42. Blockage of p38 or NF-κB pathways has shown advantages *in vitro*, but the therapeutic effect in *in vivo* experiments remains controversial. One of the reasons for this is the complex interplay between p38 and NF-κB signaling mechanisms that makes it hard to achieve the expected therapeutic effects through interventions targeting a specific pathway 43. Considering p38 and NF-κB pathways simultaneously involved in the development of VILI, one would postulate that targeting both pathways would have added benefit compared to monotherapy. Therefore, we employed SPFPT-conjugated LNPs to co-deliver Imp7 siRNA (inhibits p38 nuclear translocation) and polydatin (inhibits NF-κB). The results SPFPT@siImp7/PD-LNP simultaneously inhibited CS-induced p38 nuclear import and NF-κB activation in AECs, indicating the feasibility of the targeted delivery approach. Importantly, a more pronounced inhibition in the levels of inflammatory cytokines was observed in the siImp7 and PD codelivery group compared to the siImp or PD individual delivery group. Indeed, LNPs have been utilized for co-administrating drugs and genes, especially for cancer treatment 44.

Besides their gas exchange function, the lungs also serve as a major immune organ to protect the host from diseases caused by pathogens during respiration. AEC is an effective regulatory factor that generates primary immune responses against invading pathogens by releasing immune mediators 45. Therefore, a key issue arises: simultaneously blocking the p38 and NF-κB pathways may impair innate immunity. In addition, increasing evidence suggests that MV may sensitize the innate immune system, and conversely, the innate immune system may make the lungs sensitive to the effects of MV 46,47. Therefore, the issue of suppressing excessive inflammatory response caused by MV without affecting innate immunity needs to be addressed. In this study, we modified LNPs with activated AECs-targeting peptide and proved that the modified LNPs did not interfere with pulmonary innate immunity, as indicated by the minimal impact on the production of general recognition molecules and mediators of innate immune responses.

In summary, we developed a novel approach to identify peptides targeting activated AECs in VILI mice, by incorporating *in vivo* phage display, high-throughput sequencing, and bioinformatics analysis. The targeting peptide SPFPT was conjugated to LNPs to co-deliver siImp7 and PD. We demonstrated that the peptide-modified LNP, SPFPT@siImp7/PD-LNP, accumulated at activated AECs in VILI mice. Importantly, the SPFPT@siImp7/PD-LNP efficiently inhibited the release of inflammatory cytokines and alleviated VILI and associated distal organ injury without interfering with pulmonary innate immunity. Our study provides a novel peptide-modified LNP co-delivery strategy for the precision therapy of VILI.

## Abbreviations

AEC: alveolar epithelial cell
ALI: acute lung injury
ALT: alanine aminotransferase
ARDS: acute respiratory distress syndrome
BSA: bovine serum albumin
CE: conjugation efficiency
CS: cyclic stretch
DAO: diamine oxidase
EE: entrapment efficiency
H&E: hematoxylin and eosin
HMGB1: high mobility group box-1 protein
IL-6: interleukin-6
Imp7: importin-7
LBP: lipopolysaccharide binding protein
LNP: lipid nanoparticle
LPS: lipopolysaccharide
LTA: lipoteichoic acid
MOF: multiple organ failure
MPO: myeloperoxidase
MV: mechanical ventilation
PBS: phosphate-buffered solution
PD: Polydatin
PDI: polydispersity index
PMN: polymorphonuclear neutrophil
sCD14: soluble CD14
siRNA: small interfering RNA
TNF-α: factor-α
VILI: ventilator-induced lung injury
